# Delusion or Cultural Tradition? Diagnostic Challenges in Transcultural Psychiatry

**DOI:** 10.1192/j.eurpsy.2026.11452

**Published:** 2026-07-31

**Authors:** M. Ligero Argudo, I. M. Peso Navarro, C. García Cerdán, M. Benavides Madariaga, L. M. Domínguez-Palacios Barros, P. Salas Aranda, A. Sánchez Chillón

**Affiliations:** 1 Psychiatry; 2 Psychology, Hospital Clínico de Salamanca, Salamanca, Spain

## Abstract

**Introduction:**

Transcultural psychiatry focuses on the influence of cultural context regarding presentation, understanding and management of mental disorders. Cultural differences can represent a diagnostic challenge, especially when culturally sanctioned beliefs resemble psychotic symptoms. Misinterpretation of these experiences may lead to overdiagnosis, stigmatization, and inappropiate treatment.

**Objectives:**

To describe a case where probable psychotic symptoms were doubted and finally confirmed as culturally shared beliefs, and to discuss the implications for transcultural psychiatric practice.

**Methods:**

We describe a clinical case attended at the emergency unit of the hospital.

The patient underwent comprehensive psychiatric evaluation, including structured interviews, mental status examination, and diagnostic workup. A cultural formulation interview was used to explore explanatory models, cultural identity, and community perspectives. Family members and cultural informants were consulted to contextualize the beliefs.

**Results:**

A 29-year-old patient from Burundi with a complicated migration process is admitted to the emergency room with comportamental alterations. He presented with firm convictions of being influenced by spiritual forces, initially interpreted as delusional. Further exploration revealed these beliefs were consistent with widespread cultural traditions in the patient’s community regarding migratory processes and were not associated with disorganization, functional decline, or distress. Collateral history and a cultural mediator from the team confirmed shared cultural validity of these experiences, associated with anxiety in relation to emmigration. With psychoeducation and supportive therapy, the patient’s symptoms were reframed, avoiding unnecessary antipsychotic treatment.

**Conclusions:**

Cultural assessment in differentiating psychosis from culturally normative beliefs is essential in the therapeutic process. Cultural formulation interviews and collaboration with community informants are essential for diagnostic accuracy. Clinicians must remain cautious when interpreting unusual beliefs in culturally diverse patients, ensuring care is respectful, accurate, and patient-centered.

**Disclosure of Interest:**

None Declared

